# Mechanical Interpretation of Shrinkage Restraint in UHPC–NSC Overlays

**DOI:** 10.3390/ma19214457

**Published:** 2026-10-23

**Authors:** Duc Vinh Bui, Thuy Trang Ta

**Affiliations:** 1 Faculty of Civil Engineering, Ho Chi Minh City University of Technology (HCMUT), 268 Ly Thuong Kiet Street, Dien Hong Ward, Ho Chi Minh City 70000, Vietnam; vinhbd@hcmut.edu.vn; 2 Vietnam National University Ho Chi Minh City, Ho Chi Minh City 70000, Vietnam

**Keywords:** ultra-high-performance concrete (UHPC), shrinkage restraint, bonded overlay, normal-strength concrete, local strain measurement, partial interaction

## Abstract

Shrinkage restraint in an ultra-high-performance concrete (UHPC) overlay is commonly quantified by comparing restrained and free-shrinkage strains. However, a local restraint index may not represent the mean axial response of the overlay. This study examines the mechanical interpretation of the degree of restraint (*K_r_*) in bonded UHPC–normal-strength concrete (NSC) overlays using strain measurements in both layers. Five UHPC mixtures were monitored for 56 days at three longitudinal locations. A self-equilibrated two-layer model was used to establish a model-based restraint ceiling, *K_r_*_,max_, while apparent axial forces inferred independently from UHPC and NSC strains were compared for mechanical consistency. *K_r_*_,max_ ranged from 0.702 to 0.714. At the end of monitoring, the apparent-force ratios at midspan were 17.06 and 16.75 for 650-SFB10 and 650-SFB15, respectively, whereas the maximum for 650-SFB20 occurred at x = 900 mm (23.70). These imbalances indicate that local strains did not consistently represent mean axial strains. From approximately 14 days onward, x = 900 mm exhibited the highest Kᵣ in all five specimens. The elastic-equivalent tensile-demand ratio ranged from 1.15 to 3.06, although no visible cracking was observed. These results indicate that *K_r_* is best interpreted as a local, position- and time-dependent response index rather than a unique measure of section-level restraint.

## 1. Introduction

Ultra-high-performance concrete (UHPC) is increasingly used in thin overlays for the repair, strengthening, and protection of existing concrete structures because of its high strength and durability [1,2]. Its high binder content and low water-to-binder ratio, however, can produce substantial autogenous shrinkage, particularly at early ages [3]. When a UHPC overlay is cast on a hardened normal-strength concrete (NSC) substrate, differential shrinkage is restrained by the substrate and the interface. The resulting composite response may involve tensile and interfacial stresses, cracking or debonding, and shrinkage-induced warping, depending on the restraint configuration [4,5,6,7,8,9,10]. A meaningful assessment of restraint must therefore address not only how much shrinkage is reduced, but also what the measured strain represents mechanically.

Studies of bonded concrete overlays have established that differential-shrinkage response depends on the shrinkage of the overlay, the relative stiffness of the two layers, interfacial load transfer, and time-dependent relaxation. Silfwerbrand [4] analyzed stresses and strains in composite concrete beams, whereas Beushausen and Alexander [5,6] examined failure mechanisms, tensile relaxation, and localized strain and stress in bonded overlays. Zhou et al. [7] developed a plate-based model incorporating an interfacial shear stress–slip relation and investigated the effects of interface shear stiffness, repair-system dimensions, and elastic moduli. For UHPC-based systems, Zhu et al. [8] studied shrinkage-induced restrained stresses in UHPC–RC composites, Teng et al. [9] developed low-shrinkage UHPC for thin bonded overlays, and Guo et al. [10] examined shrinkage-induced warping of UHPC overlays cast on hardened NSC. More recent UHPC studies have highlighted the roles of relative UHPC–NSC stiffness and time-dependent creep/relaxation in restrained shrinkage and stress development [11,12]. Bond studies have also shown that surface condition, surface roughness, and interfacial behavior influence composite action [2,13,14,15,16,17], while interfacial transition-zone characteristics have been linked to bond performance in UHPC repair systems [18]. Liu et al. [19] related restrained shrinkage to cracking and delamination in UHPC–concrete composite slabs.

The degree of restraint, *K_r_*, is commonly obtained by comparing the strain of a restrained overlay with its free-shrinkage strain. This measure is useful for describing the observed reduction in shrinkage, but its mechanical interpretation is less straightforward when a strain recorded at a single location is treated as the mean axial strain of the overlay. Differential shrinkage in a one-sided overlay can produce axial deformation and curvature simultaneously, resulting in a through-depth strain gradient and making the measurement depth relevant [6,7,10]. Interfacial shear transfer and partial interaction can further modify the local strain field [20,21]. Recent studies have demonstrated the sensitivity of UHPC–NSC overlay response to restraint configuration [22], while interfacial slip can affect restrained-stress estimates [23]. Structural details, including overlay connection and construction configurations, have also been investigated in UHPC–NSC overlay systems [24,25]. These developments make it important to distinguish spatial variation itself from the mechanical meaning assigned to a locally measured strain.

Despite this progress, it remains unclear whether local strains measured simultaneously in the overlay and substrate at different longitudinal locations can be treated as the section-level axial strains required by a self-equilibrated two-layer model. This distinction matters because a local measurement may contain contributions from axial restraint, section curvature, measurement eccentricity, partial interaction, and boundary-sensitive deformation. Consequently, agreement between restrained and free-shrinkage measurements alone does not establish that the measured strain is representative of the mean axial response of either layer. A direct check requires strain information from both sides of the composite system and a mechanical reference against which those measurements can be tested.

To examine this issue, simultaneous strain measurements in the UHPC overlay and NSC substrate were combined with independently measured free shrinkage and a self-equilibrated axial reference. The measured local strains were then assessed against the restraint level and internal-force compatibility implied by this reference. An elastic-equivalent tensile-demand ratio was also used as an additional reference for interpreting the measured UHPC strain. These comparisons were intended as mechanical consistency tests rather than as estimates of the actual time-dependent stress state.

Two questions are addressed. First, what reference level of restraint follows from the relative axial stiffness of the UHPC overlay and NSC substrate under a self-equilibrated elastic model with no external axial load? Second, are the local strains measured in the UHPC and NSC sufficiently representative of mean axial strain to support internal-force inference? The consistency of this interpretation is examined at three longitudinal locations to assess its sensitivity to specimen boundaries. The midspan station is used as the primary comparison location because it is the most interior measurement position, not because it is assumed to be unaffected by boundary conditions.

The contribution of this study is an experimental–mechanical consistency check that uses simultaneous measurements in both layers to distinguish the local restraint response captured by the sensors from the section-level axial response implied by a simplified self-equilibrated reference. This distinction provides a direct basis for judging when a locally measured shrinkage strain should not be used as a section-representative axial strain for internal-force inference.

## 2. Mechanical Framework for Interpreting Shrinkage Restraint

This section establishes a mechanical reference framework for interpreting strain measurements in UHPC–NSC overlays. The framework is not intended to reproduce the complete time-dependent response of the composite system. Instead, it provides two consistency checks that can be evaluated directly from the measurements: (i) a reference restraint level for a self-equilibrated two-layer system and (ii) a test of whether locally measured strains can reasonably represent the mean axial strains required for internal-force inference. The formulation draws on differential-shrinkage mechanics for bonded overlays [4,5,6,7], restrained shrinkage and warping in UHPC–concrete composites [8,10,19,22,23,24,25], and partial-interaction mechanics [20,21].

### 2.1. Response Quantities

The measured local longitudinal strains in the UHPC overlay and NSC substrate are denoted by *ε*_1_(*x*,*t*) and *ε*_2_(*x*,*t*), respectively, where *x* is the measurement position and *t* is time. The free-shrinkage strain measured on a separate UHPC specimen is denoted by *ε*_free_(*t*). The local strain difference between the two layers and the degree of restraint of the overlay are then defined as:

∆*ε*_12_(*x*,*t*) = |*ε*_1_(*x*,*t*) − *ε*_2_(*x*,*t*)| (1)

(2) $$K_{r}\left(x,t\right)=1-\frac{\left|\varepsilon_{1}(x,t)\right|}{\left|\varepsilon_{\mathrm{free}}(t)\right|}$$

where ∆*ε*_12_(*x*,*t*) represents the local strain difference between the UHPC overlay and NSC substrate at the corresponding measurement position. Since the two strains are measured at different depths within the composite section, ∆*ε*_12_ may include a contribution associated with section curvature and is therefore not equivalent to interfacial slip. The strain-based degree of restraint, *K_r_*(*x*,*t*), quantifies the reduction in the measured UHPC shrinkage strain relative to the free-shrinkage strain, with *K_r_* = 0 corresponding to the free-shrinkage reference and increasing *K_r_* indicating a greater reduction in the measured shrinkage strain.

### 2.2. Self-Equilibrated Axial Reference and Model-Based Restraint Ceiling

For the present 400 mm specimen width, the axial areas are A_1_ = 400 × 50 = 20,000 mm^2^ for UHPC and A_2_ = 400 × 200 = 80,000 mm^2^ for NSC. Using the 28-day moduli (UHPC: 48.1–50.9 GPa; NSC: 30.0 GPa), consistently expressed in MPa, yields the dimensionless reference ratio in Equation (8).

The reference model idealizes the UHPC overlay (layer 1) and NSC substrate (layer 2) as two axial members coupled through an interface capable of transferring shear. The formulation assumes that (i) no external axial force acts on the composite section; (ii) the UHPC is subjected to the free-shrinkage eigenstrain *ε*_free_; (iii) shrinkage developing in the mature NSC substrate during the monitoring period is small relative to that of the newly cast UHPC; (iv) each layer is represented by a mean axial strain and its measured elastic modulus; and (v) the effects of curvature, interfacial slip, creep and relaxation, and support restraint are neglected. The NSC substrate was 150 days old when the UHPC overlay was cast. The mature substrate was selected to limit the influence of ongoing NSC shrinkage on the measured differential response, rather than to assume that substrate shrinkage had ceased. Residual NSC shrinkage is therefore treated as a secondary contribution to the differential-shrinkage response [4,5]. Since the free-shrinkage history of the NSC substrate was not measured independently, this contribution cannot be separated quantitatively from the measured response. The resulting formulation is used as a mechanical reference for interpreting the experimental strains, rather than as a complete representation of the time-dependent behavior of the bonded UHPC–NSC system [4,5,6,7,8,10].

(3) $$N_{1}=A_{1}E_{1}\left({\bar{\varepsilon }}_{1}-\varepsilon_{\mathrm{free}}\right),\;N_{2}=A_{2}E_{2}{\bar{\varepsilon }}_{2}$$

*N*_1_ + *N*_2_ = 0 (4)

where *N_i_*, *A_i_*, *E_i_*, and ${\bar{\varepsilon }}_{i}$ denote the axial force, cross-sectional area, elastic modulus, and mean axial strain of layer *i*, respectively, with *i* = 1 for the UHPC overlay and *i* = 2 for the NSC substrate. Unlike the local measured strains *ε*_1_(*x*,*t*) and *ε*_2_(*x*,*t*) defined in Section 2.1, ${\bar{\varepsilon }}_{1}$ and ${\bar{\varepsilon }}_{2}$ represent the mean axial strains required by the reference model.

For the limiting axial reference considered here, full axial compatibility is additionally imposed, such that the two layers share a common mean axial strain:

(5) $${\bar{\varepsilon }}_{1}{=\bar{\varepsilon }}_{2}=\varepsilon_{c}$$

where *ε_c_* denotes the common mean axial strain of the two layers. Substitution of Equation (5) into Equations (3) and (4) gives:

*A*_1_*E*_1_(*ε_c_* − *ε*_free_)+*A*_2_*E*_2_*ε_c_* = 0 (6)

and therefore

(7) $$\varepsilon_{c}=\frac{A_{1}E_{1}}{A_{1}E_{1}+A_{2}E_{2}}\varepsilon_{\mathrm{free}}$$

The corresponding model-based restraint ceiling, *K_r_*_,max_, is

(8) $$K_{r,\max}=\frac{A_{2}E_{2}}{A_{1}E_{1}+A_{2}E_{2}}=\frac{1}{1+A_{1}E_{1}/{(A}_{2}E_{2})}<1$$

With finite interfacial stiffness, axial force may develop from a free edge toward the interior through interfacial shear transfer, as described in bonded-overlay and partial-interaction formulations [4,6,7,20,21]. UHPC–NSC studies likewise show that surface preparation and bond characteristics can govern load transfer across the interface [2,13,14,15,16,17]. Interface shear tests further show that substrate strength and surface roughness affect shear resistance and load–slip response [26], while interfacial slip can affect the interpretation of restrained stress [23]. No independent interface stiffness is calibrated here because direct slip or bond–slip measurements were not available. The model-based ceiling is therefore used as a mechanical reference rather than as a complete predictive description of the bonded overlay.

Equation (8) defines an upper bound for *K_r_* only within this self-equilibrated axial reference; it is not a universal limit for UHPC–NSC overlays. The bound follows from stiffness equilibrium and full axial compatibility and is not fitted to the measurements. The measured elastic moduli are used as fixed reference stiffnesses, so *K_r_*_,max_ does not represent a time-dependent restraint prediction incorporating *E*(*t*), creep/relaxation, curvature, or partial interaction. Recent restrained-stress modeling of UHPC has likewise shown the importance of accounting for in-situ creep in stress prediction [27]. A sustained value above *K_r_*_,max_ indicates that the axial reference does not fully represent the measured response, but the exceedance alone does not identify the mechanism responsible for the discrepancy.

### 2.3. Bending and Sensor Eccentricity

The reference model in Section 2.2 is formulated in terms of the mean axial strain of each layer. However, a one-sided overlay subjected to differential shrinkage can develop both axial deformation and curvature. Previous studies involving localized strain measurements [6], plate/interface-shear formulations [7], partial-interaction mechanics [20,21], and UHPC–NSC warping [10] indicate that a strain measured at a specific depth need not represent the mean axial strain of the corresponding layer. In the present specimens, the sensors were located eccentrically with respect to the centroids of both layers. Consequently, forces inferred directly from the measured local strains are treated here as apparent forces for assessing consistency with the axial reference model, rather than as direct measurements of internal axial force.


(9) $$\varepsilon_{i}\left(z,x,t\right)={\bar{\varepsilon }}_{i}\left(x,t\right)+\kappa \left(x,t\right)z$$

In Equation (9), *ε_i_*(*z*,*x*,*t*) is the local longitudinal strain in layer *i* at position *x* and time *t*; ${\bar{\varepsilon }}_{i}(x,t)$ is the mean axial strain of layer *i*; *κ*(*x*,*t*) is the curvature; and *z* is the signed distance from the centroidal axis of layer *i* to the measurement location. Thus, for an eccentric sensor (*z* ≠ 0), the measured local strain may contain both axial and bending contributions. Direct substitution of the measured local strains *ε*_1_(*x*,*t*) and *ε*_2_(*x*,*t*) into the axial force relations of the reference model therefore provides apparent, rather than actual, axial forces:

(10) $$N_{1}^{*}\left(x,t\right)=A_{1}E_{1}\left[\varepsilon_{1}\left(x,t\right)-\varepsilon_{\mathrm{free}}\left(t\right)\right],\;N_{2}^{*}\left(x,t\right)=A_{2}E_{2}\varepsilon_{2}(x,t)$$

where $N_{1}^{*}(x,t)$ and $N_{2}^{*}(x,t)$ denote the apparent axial forces inferred from the measured local strains in the UHPC overlay and NSC substrate, respectively. If *ε*_1_(*x*,*t*) and *ε*_2_(*x*,*t*) adequately represent the mean axial strains of the two layers, the apparent forces in Equation (10) should approximately satisfy $N_{1}^{*}\left(x,t\right)+N_{2}^{*}\left(x,t\right)=0$, consistent with the equilibrium condition in Equation (4). Conversely, a systematic imbalance between $N_{1}^{*}\left(x,t\right)$ and $N_{2}^{*}\left(x,t\right)$ indicates that direct conversion of the measured local strains into axial forces is not mechanically consistent with the assumptions of the reference model. Such an imbalance does not imply a violation of global force equilibrium; rather, it indicates that the measured local strains cannot, in general, be interpreted as section-representative mean axial strains without considering effects omitted from the reference model, including curvature, sensor eccentricity, partial interaction, and boundary restraint.

### 2.4. Mechanical Consistency Criteria and Treatment of Boundary Effects

Two mechanical consistency criteria are used to assess the measured response against the axial reference model. First, under the self-equilibrated axial reference defined in Section 2.2, *K_r_* should not exceed the model-based ceiling *K_r_*_,max_ given by Equation (8). A sustained exceedance indicates that the measured response is not fully described by the reference model. The exceedance alone, however, does not identify the mechanism responsible for the discrepancy.

Second, if the local strains measured in the two layers adequately represent their respective mean axial strains, the apparent forces calculated from Equation (10) should approximately satisfy $N_{1}^{*}+N_{2}^{*}=0$, consistent with Equation (4). A systematic mismatch between $N_{1}^{*}$ and $N_{2}^{*}$ indicates that direct conversion of the measured local strains into axial forces is not consistent with the assumptions of the reference model. Variation in this mismatch among measurement locations is used to assess the spatial dependence of this consistency.

**Figure 1:**
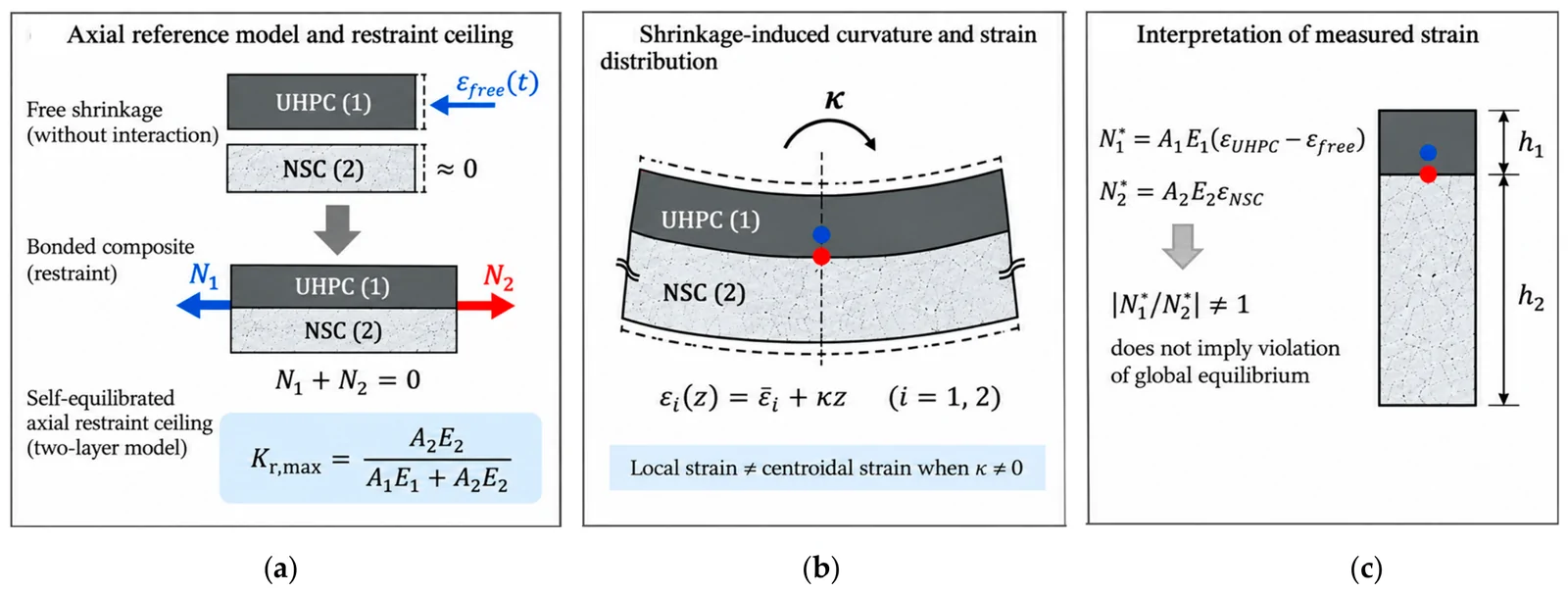
Mechanical interpretation of shrinkage restraint in UHPC–NSC overlays: (**a**) self-equilibrated axial reference and model-based restraint ceiling; (**b**) shrinkage-induced curvature and strain distribution; (**c**) interpretation of measured local strains.

To further examine the mechanical implication of the measured UHPC strain, an elastic-equivalent tensile-demand ratio is defined as

(11) $$R_{t,\mathrm{el}}\left(x,t\right)=\frac{E_{1}\left|\varepsilon_{1}\left(x,t\right)-\varepsilon_{\mathrm{free}}(t)\right|}{f_{t,\mathrm{peak}}}$$

where *E*_1_ is the measured elastic modulus of the UHPC mixture and *f_t_*_,peak_ is its measured peak direct tensile strength. The numerator represents an elastic-equivalent tensile demand obtained by directly converting the difference between the measured local UHPC strain and the free-shrinkage strain into stress using *E*_1_. Accordingly, *R_t_*_,el_ is used as a normalized reference quantity and is not interpreted as a measure of the actual tensile stress in the overlay. A value of *R_t_*_,el_ > 1 indicates that the elastic-equivalent tensile demand exceeds the measured peak direct tensile strength; it does not, by itself, indicate cracking or failure. The mechanical framework used to interpret the local strain measurements, including the axial reference and the possible contribution of curvature, is summarized schematically in Figure 1. This elastic-equivalent construction is conceptually consistent with restrained-concrete approaches that compare a theoretical elastic stress derived from free shrinkage with the residual stress to assess stress relaxation [28].

Sensitivity to specimen boundaries is examined by comparing the measured response at the three longitudinal stations. A global longitudinal coordinate *x* is defined from the left edge of the 1200 mm specimen, with measurement stations at *x* = 100, 600, and 900 mm. Two 150 × 150 × 150 mm concrete supports are positioned symmetrically near the specimen ends, with the outer edge of each support located 125 mm from the corresponding slab edge. The left support therefore occupies *x* = 125–275 mm and the right support *x* = 925–1075 mm. The *x* = 100 mm station is located 25 mm outside the left support-contact region, whereas the *x* = 900 mm station is located 25 mm toward the specimen interior from the inner edge of the right support-contact region. The *x* = 600 mm station is at midspan and is 325 mm from the inner edge of either support-contact region. As the farthest measured station from both free edges and support-contact regions, *x* = 600 mm is used as the primary location for comparison with the axial reference model. This selection is based on geometry and does not imply that the midspan response is unaffected by the specimen boundaries. The two off-center stations are retained to assess the spatial variation in the measured response near the edge and support regions.

Together, the restraint-ceiling and apparent-force checks assess the consistency of the measured local strains with the section-level assumptions of the axial reference model, while *R_t_*_,el_ provides a normalized comparison between the elastic-equivalent tensile demand and the measured tensile strength. Differences among the three measurement stations are interpreted as spatial variation that may reflect boundary influence, without attributing the observed response to a specific unmeasured mechanism such as support friction.

## 3. Materials and Methods

### 3.1. Materials, Mixture Design, and Mechanical Properties

Five UHPC mixtures were investigated. Four mixtures contained 650 kg/m^3^ of cement (650-SFB00, 650-SFB10, 650-SFB15, and 650-SFB20), with steel-fiber volume fractions of 0, 1.0, 1.5, and 2.0%, respectively. The paste composition was identical within this series, while the aggregate proportions were adjusted to accommodate the different fiber contents (Table 1). The fifth mixture, 740-SFB00, was fiber-free and contained a higher cement content. Fiber-related trends are therefore evaluated only within the 650 series, while 740-SFB00 is considered separately. The mixture proportions and measured mechanical properties are summarized in Table 1 and Table 2.

The maximum particle size of the crushed aggregate was approximately 2.5 mm. The decrease in coarse-aggregate mass from 703 to 401 kg/m^3^ within the 650 series reflects adjustments to the aggregate skeleton as fiber volume increased. Straight, copper/brass-coated steel fibers (OL FIBER WSF020-13, LANMIX Fiber Vietnam) were 13 mm long and 0.20 mm in diameter (aspect ratio 65), with a nominal density of 7800 kg/m^3^ and a manufacturer-reported tensile strength of 2800 MPa (±7.5%). Fiber shape, aspect ratio, and dosage may influence fiber bridging and the mechanical response [29].

The compressive elastic modulus of each UHPC mixture was determined from five specimens and reported as the mean. The NSC elastic modulus was similarly determined from five specimens cast from the same batch, yielding a mean elastic modulus of 30.00 GPa; the corresponding compressive strength was 38 MPa. The UHPC compressive elastic moduli and peak direct tensile strengths were determined at 28 days (*n* = 5 per mixture); the NSC elastic modulus was also measured at 28 days (*n* = 5; SD 1.355 GPa). The 28-day compressive elastic modulus, rather than a directly measured tensile modulus, was used as the fixed stiffness reference in the elastic-equivalent tensile-demand calculation. These 28-day values do not describe the age-dependent stiffness throughout the monitoring period.

**Figure 2:**
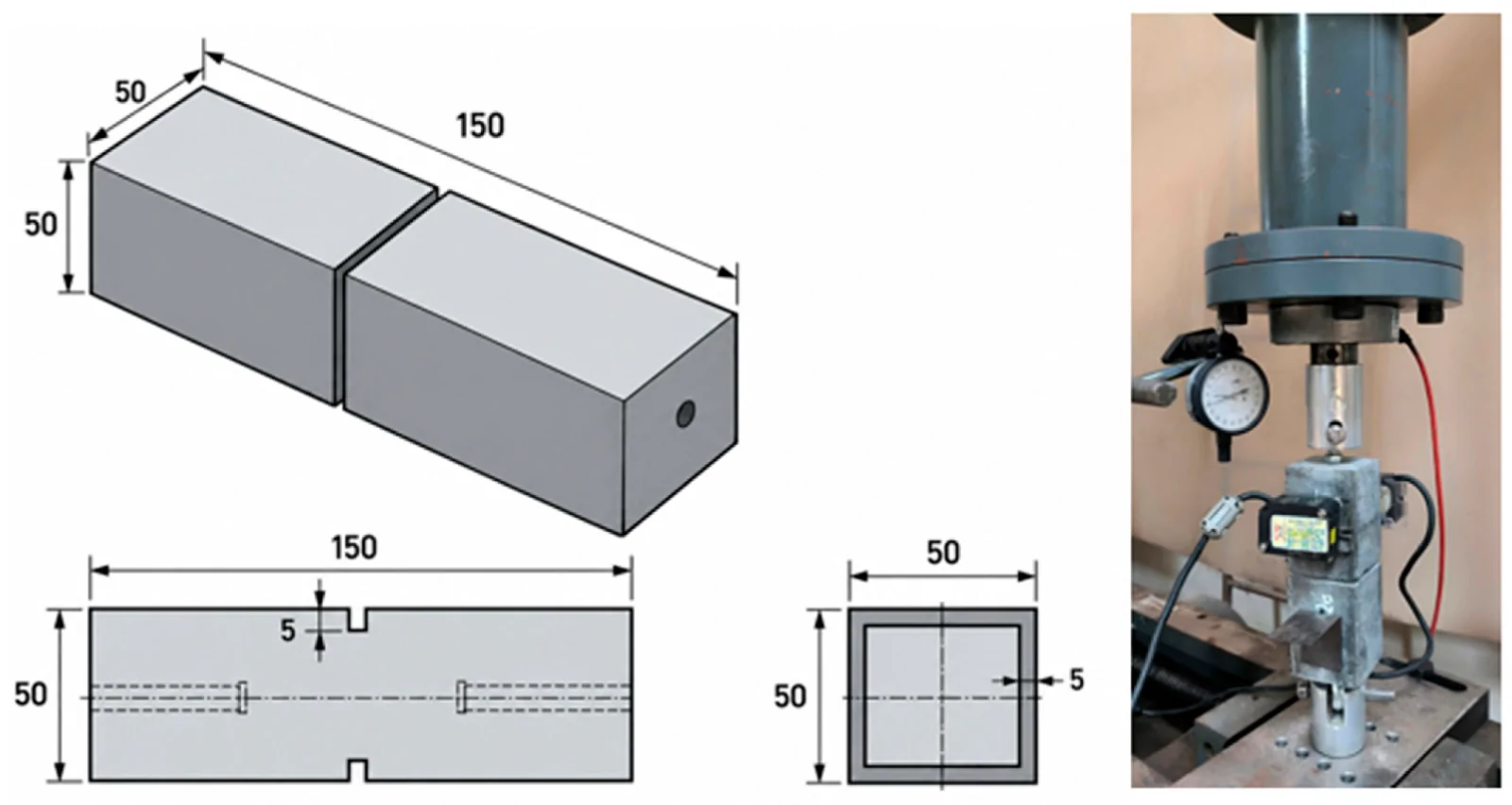
Direct tensile test of UHPC: test setup and specimen geometry (50 × 50 × 150 mm), with 5 mm deep notches on all four faces.

The direct tensile strength of UHPC was measured using 50 × 50 × 150 mm prismatic specimens. At mid-length, 5 mm deep notches were cut on all four faces, reducing the net load-bearing section to 40 × 40 mm (1600 mm^2^) and promoting failure within the notched region. The specimens were loaded axially in tension using a dedicated test frame. Opening across the notched region was monitored by two symmetrically arranged laser sensors and independently checked using a mechanical dial gauge with a resolution of 1 µm. Load and displacement were recorded simultaneously using a digital data-acquisition system. All specimens failed within the notched region. The peak direct tensile strength, *f_t_*_,peak_, was calculated as the maximum tensile load divided by the net cross-sectional area at the notch. The test setup and specimen geometry are shown in Figure 2.

### 3.2. Preparation of the NSC Substrate and UHPC–NSC Composite Specimens

**Figure 3:**
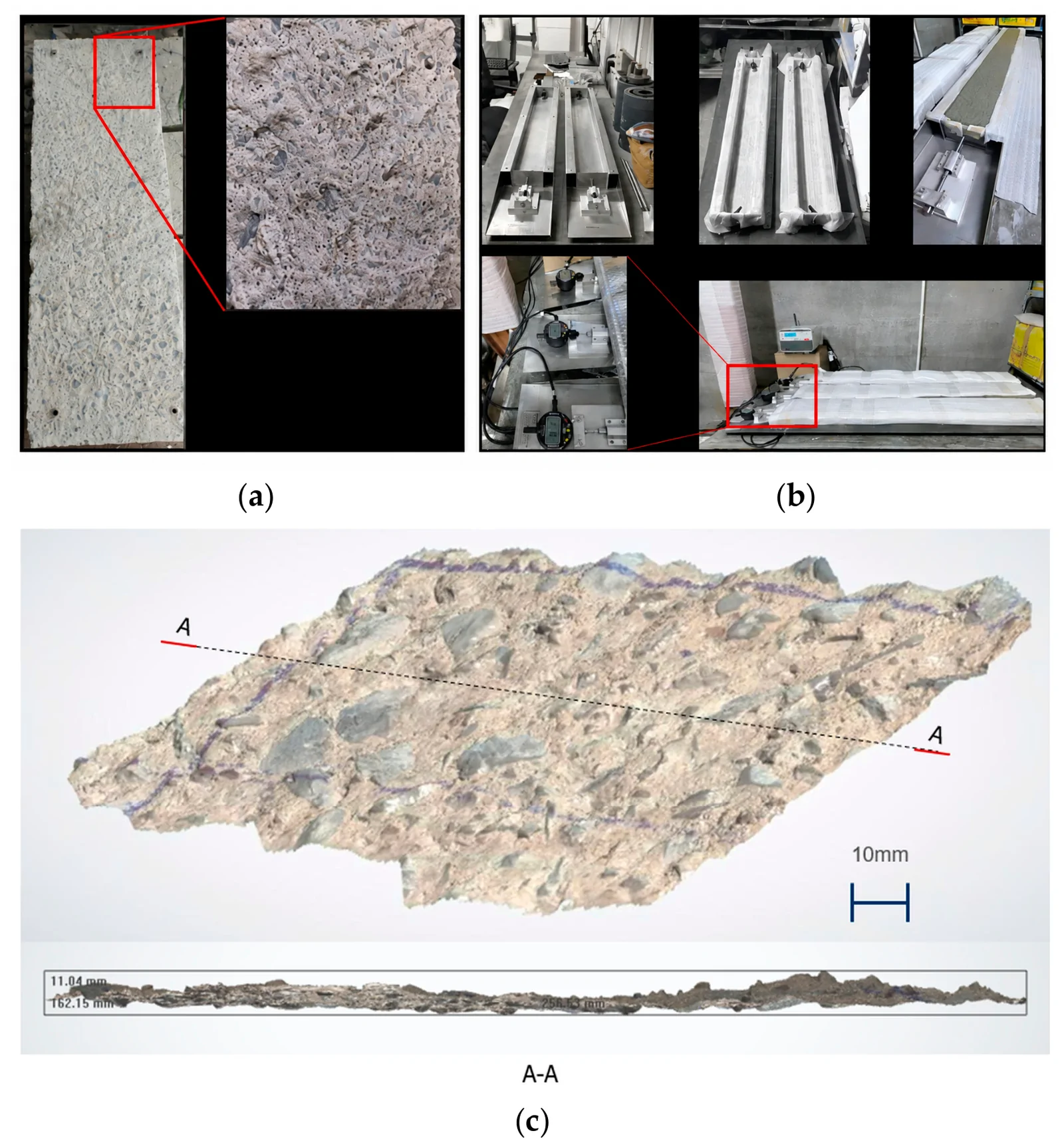
Experimental components and surface characterization: (**a**) NSC surface after mechanical roughening; (**b**) UHPC free-shrinkage specimen and measurement system; (**c**) 3D scan of the prepared NSC surface and corresponding A–A section profile.

The NSC substrate was cast in advance and was 150 days old when the UHPC overlay was placed. Using a mature substrate was intended to reduce, rather than eliminate, the contribution of ongoing NSC shrinkage during monitoring. Because a companion free-shrinkage specimen was not monitored for the NSC, residual substrate shrinkage could not be independently quantified and remains an acknowledged simplification of the reference model. Before UHPC casting, the NSC surface was mechanically roughened and thoroughly cleaned to remove loose particles and provide a suitable bonding surface for the overlay. The prepared NSC surface is shown in Figure 3a, while the UHPC free-shrinkage specimen and measurement system are shown in Figure 3b. The prepared surface was further characterized by three-dimensional (3D) scanning (Figure 3c), providing a geometric representation of the surface condition prior to overlay placement. The resulting 3D surface model and corresponding section profile indicated local surface-relief depths of approximately 3–8 mm over the scanned area. This characterization was used to document the prepared interface geometry rather than to derive a quantitative bond or interface-stiffness parameter for the mechanical analysis.

One composite specimen was prepared for each UHPC mixture, giving five UHPC–NSC composite specimens in total. Each specimen was 1200 mm long and 400 mm wide and consisted of a 200 mm thick NSC substrate and a 50 mm thick UHPC overlay, resulting in a total composite thickness of 250 mm. The UHPC was cast directly onto the prepared NSC surface to form the bonded overlay system. The same specimen geometry and support arrangement were maintained for all five mixtures so that differences in the measured response could be examined under a common test configuration. Two 150 × 150 × 150 mm concrete blocks were positioned symmetrically near the specimen ends to support the composite specimen during monitoring. The outer edge of each support was located 125 mm from the corresponding specimen edge. Using a global longitudinal coordinate *x* measured from the left edge of the specimen, the left and right support-contact regions therefore occupied *x* = 125–275 mm and *x* = 925–1075 mm, respectively. This coordinate system is also used to define the longitudinal strain-measurement locations described in the following section.

### 3.3. Strain and Free-Shrinkage Measurements

**Figure 4:**
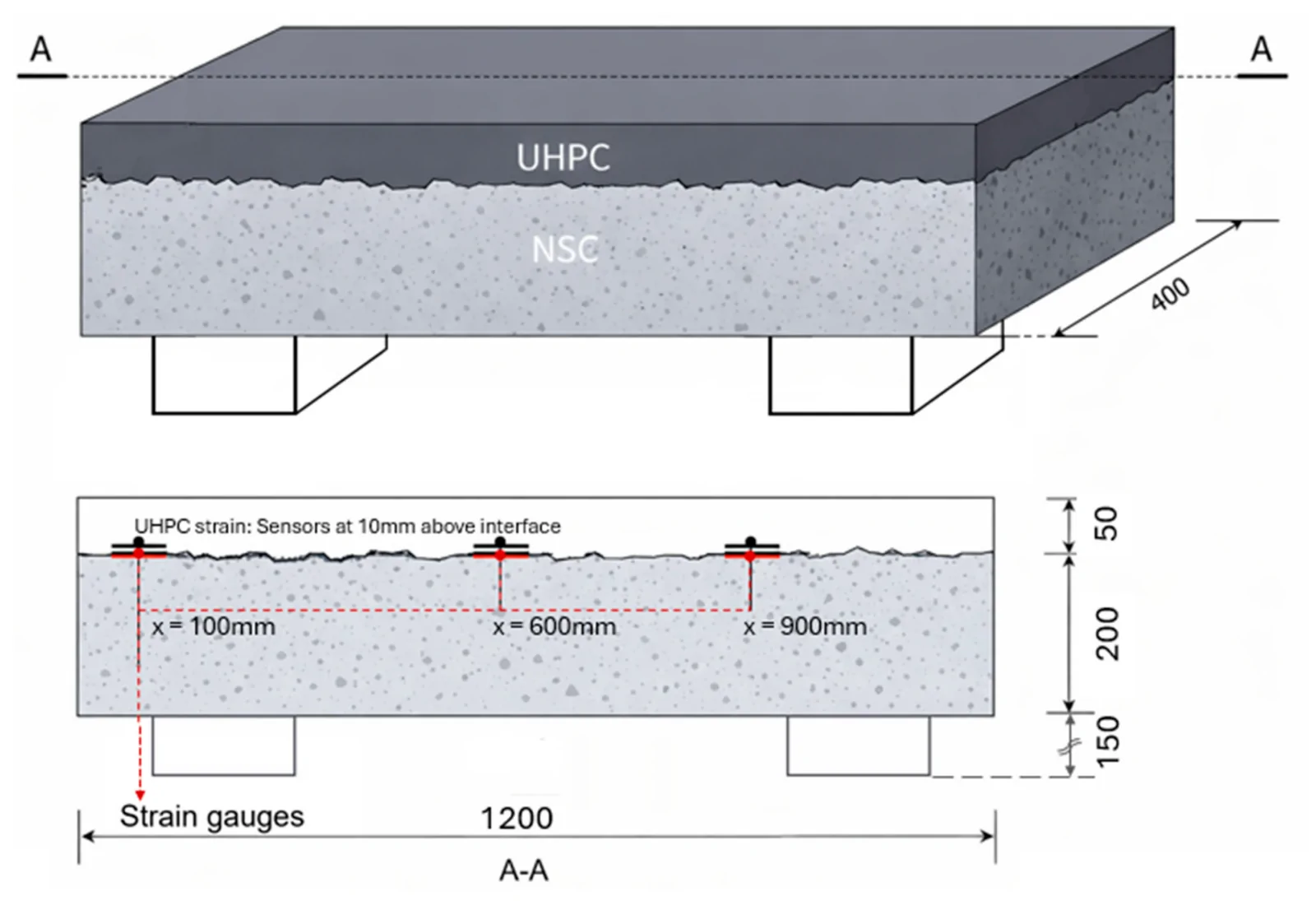
Experimental configuration and geometry of the UHPC–NSC composite specimen.

Strains in the UHPC overlay and NSC substrate were recorded simultaneously at three longitudinal stations. Using the global coordinate *x* defined in Section 3.2, the stations were located at *x* = 100, 600, and 900 mm, corresponding respectively to 100 mm from the left end, the specimen midspan, and 300 mm from the right end. Relative to the support-contact regions, the *x* = 100 mm station was located 25 mm outside the left support, the *x* = 900 mm station was located 25 mm toward the specimen interior from the inner edge of the right support, and the *x* = 600 mm station was 325 mm from the inner edge of either support. The composite specimen geometry, support arrangement, and measurement stations are shown in Figure 4.

**Figure 5:**
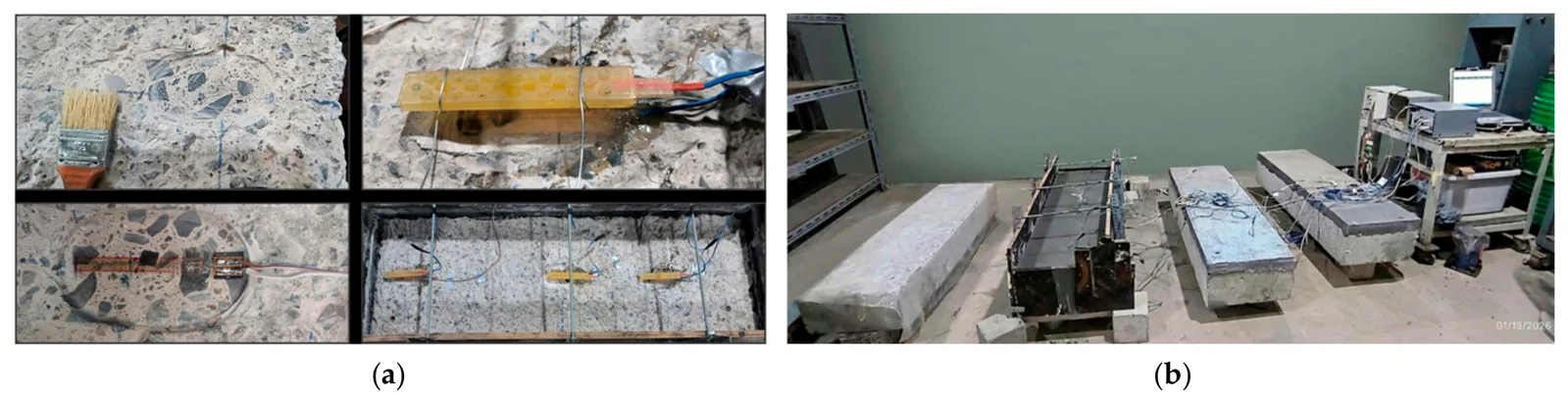
Strain measurement in the composite test: (**a**) installation and protection of strain sensors; (**b**) UHPC–NSC specimen and data-acquisition system.

At each station, the UHPC sensor was positioned 10 mm above the UHPC–NSC interface, corresponding to an eccentricity of 15 mm from the centroid of the 50 mm thick overlay. The NSC sensor was positioned at the UHPC–NSC interface, approximately 100 mm from the centroid of the 200 mm thick substrate. The strain-sensing system used TML (Tokyo Measuring Instruments Laboratory) PL-60-11, 120 Ω strain gauges protected within an epoxy-resin body for embedment. The PL-60-11 gauges were self-temperature-compensated for a nominal linear expansion coefficient of approximately 11 × 10^−6^/°C, reducing apparent strain associated with temperature variations. The protected sensor assembly had an approximately 60 mm sensing region, an overall protected length of approximately 115 mm, and an encapsulated section approximately 15 mm wide and 4 mm thick. The protected sensors, their installation, and the composite monitoring system are shown in Figure 5. Because only one measurement depth was available in each layer, the recorded strains are treated as local strains at the sensor locations and are not assumed to equal the mean axial strains of the respective layers.

The free-shrinkage strain, *ε*_free_, of each UHPC mixture was measured independently on a separate specimen using the long-trough configuration shown in Figure 3b. Each mixture therefore had one composite specimen and one corresponding free-shrinkage specimen. The experimental design was intended to assess the mechanical consistency framework separately for each mixture–specimen case rather than to provide a statistical comparison among mixtures. The composite and free-shrinkage specimens were monitored under the same indoor laboratory conditions at a temperature of 27 ± 1 °C and a relative humidity of approximately 60%. Strain monitoring continued for 56 days for all mixtures. A common zero time of 6 h after mixing was adopted for the embedded-gauge and free-shrinkage measurements; strains in both systems were referenced to their readings at that time. Thus, deformation before 6 h is excluded and early-age restraint indices may depend on this reference choice.

Prior to the mechanical analysis, the measured strain histories were processed to characterize their long-term evolution. The free-shrinkage histories were smoothed using a centered moving average with an approximately one-day window to reduce short-term fluctuations. For the UHPC and NSC measurements, short-term fluctuations were superimposed on the longer-term strain trends. A Weibull-type exponential function was therefore used to represent the central and later-age trends of the UHPC and NSC strain histories. This approach reduced the influence of short-term fluctuations while retaining the overall evolution of the measured strains.

The processed strain histories were subsequently used to calculate the local strain difference (Δε_12_), degree of restraint (*K_r_*), apparent axial forces ($N_{1}^{*}$ and $N_{2}^{*}$), apparent-force ratio (*R*_F_), and elastic-equivalent tensile-demand ratio (*R_t_*_,el_). For reproducibility, the original Weibull-type function used to represent the 30 UHPC and NSC strain histories is expressed as:

(12) $$\varepsilon_{\mathrm{fit}}\left(t\right)=A(1-e^{bt^{c}})$$

where *t* is the time in days, *A* and *b* are signed fitting parameters (both negative for the fitted shrinkage histories), and *c* > 0. The fitting parameters were selected to represent the central and later-age strain trends rather than obtained by minimizing an unweighted residual sum of squares over all measurement times. Accordingly, the fitted functions are used as empirical representations of the measured strain trends rather than as constitutive shrinkage laws. Exceedance fractions calculated from the fitted strain histories are reported as fit-derived values rather than direct counts of the original sensor measurements.

### 3.4. Data Processing and Response Evaluation

The mechanical indices were evaluated separately at the three measurement stations (x = 100, 600, and 900 mm) to preserve the spatial variation in the measured response. As an independent end-of-monitoring sensitivity check, the original sensor strain records were also used to calculate the indices at a common age of 56.00 days, using linear interpolation between adjacent measurements where necessary. This comparison was performed to assess the sensitivity of the end-of-monitoring interpretation to the use of fitted strain histories. The original Weibull-type trend curves were retained for the main analysis. The original Weibull-type fitting parameters (A, b, and c) and the corresponding reported R^2^ values for all 30 UHPC and NSC strain histories are summarized in Table 3.

The local strain difference, Δ*ε*_12_, and degree of restraint, *K_r_*, were calculated using Equations (1) and (2), respectively. Δ*ε*_12_ was treated as a local strain-difference index and not as interfacial slip. The measured *K_r_* was compared with the model-based restraint ceiling *K_r_*_,max_ defined in Equation (8). Because *K_r_* contains ∣*ε*_free_∣ in the denominator, this comparison was restricted to *t* ≥ 1 day to avoid the small-denominator region at very early ages. For reporting and comparison in the Results, the magnitude |Δε_12_| = |ε_1_ − ε_2_| is used; the sign of Equation (1) is therefore not used to distinguish the direction of the local strain difference. The absolute-value convention in Equation (2) compares shrinkage magnitudes without assigning tensile/compressive sign to the index; the signed strain histories remain available for interpretation. The index is not evaluated where the free-shrinkage magnitude is close to zero or during local sign reversals, because the ratio can become unstable.

The apparent axial forces $N_{1}^{*}$ and $N_{2}^{*}$ were calculated using Equation (10) to assess the consistency of interpreting the measured local strains as mean axial strains. The elastic-equivalent tensile-demand ratio *R_t_*_,el_ was calculated using Equation (11), with the measured elastic modulus and peak direct tensile strength of each mixture used as fixed reference values. Because the age-dependent evolution of these properties was not continuously measured, *R_t_*_,el_ was evaluated primarily at the end of monitoring.

## 4. Results

### 4.1. Free Shrinkage and Direct Tensile Strength of UHPC

**Figure 6:**
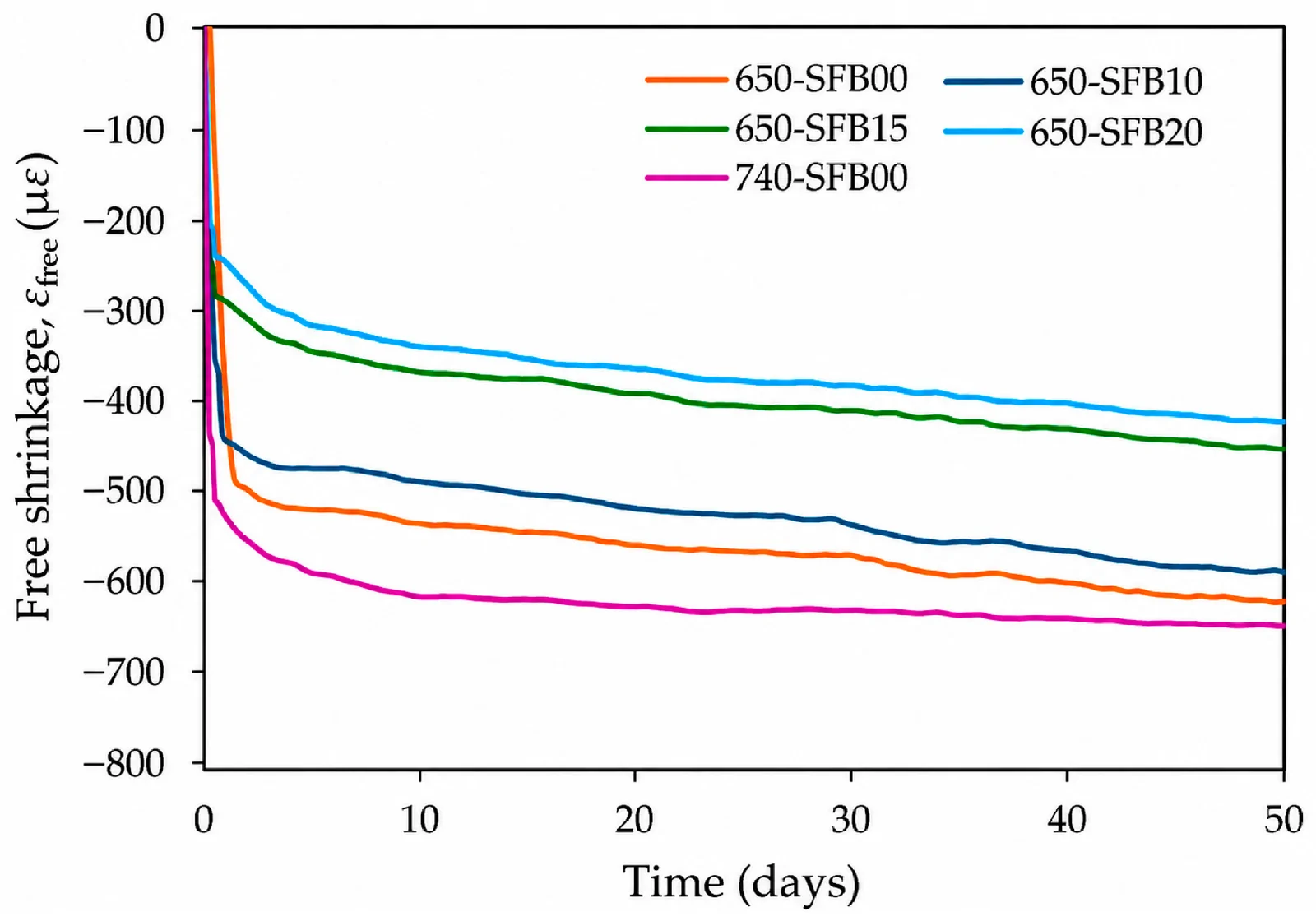
Processed free-shrinkage histories of the five UHPC mixtures using a centered moving average with an approximately one-day window.

The five mixtures exhibited different free-shrinkage histories. At the end of monitoring, *ε*_free_ reached approximately −616.0, −583.0, −460.0, −427.0, and −649.0 µε for 650-SFB00, 650-SFB10, 650-SFB15, 650-SFB20, and 740-SFB00, respectively. Within the 650 series, which had an identical paste composition but adjusted aggregate proportions as fiber content increased, the end-of-monitoring free shrinkage decreased progressively across the tested specimens with increasing nominal fiber content. The processed free-shrinkage histories used in the subsequent analysis are shown in Figure 6.

The measured peak direct tensile strengths, *f_t_*_,peak_, were 5.49, 7.86, 7.91, 8.78, and 6.00 MPa for 650-SFB00, 650-SFB10, 650-SFB15, 650-SFB20, and 740-SFB00, respectively. These values were used as the reference tensile strengths in calculating *R_t_*_,el_. Within the 650 series, the specimens with higher fiber contents showed both lower end-of-monitoring free shrinkage and higher peak direct tensile strength. These measured quantities provide the strain and strength references, respectively, for the subsequent mechanical assessment.

### 4.2. Measured Strain Difference Between UHPC and NSC

**Figure 7:**
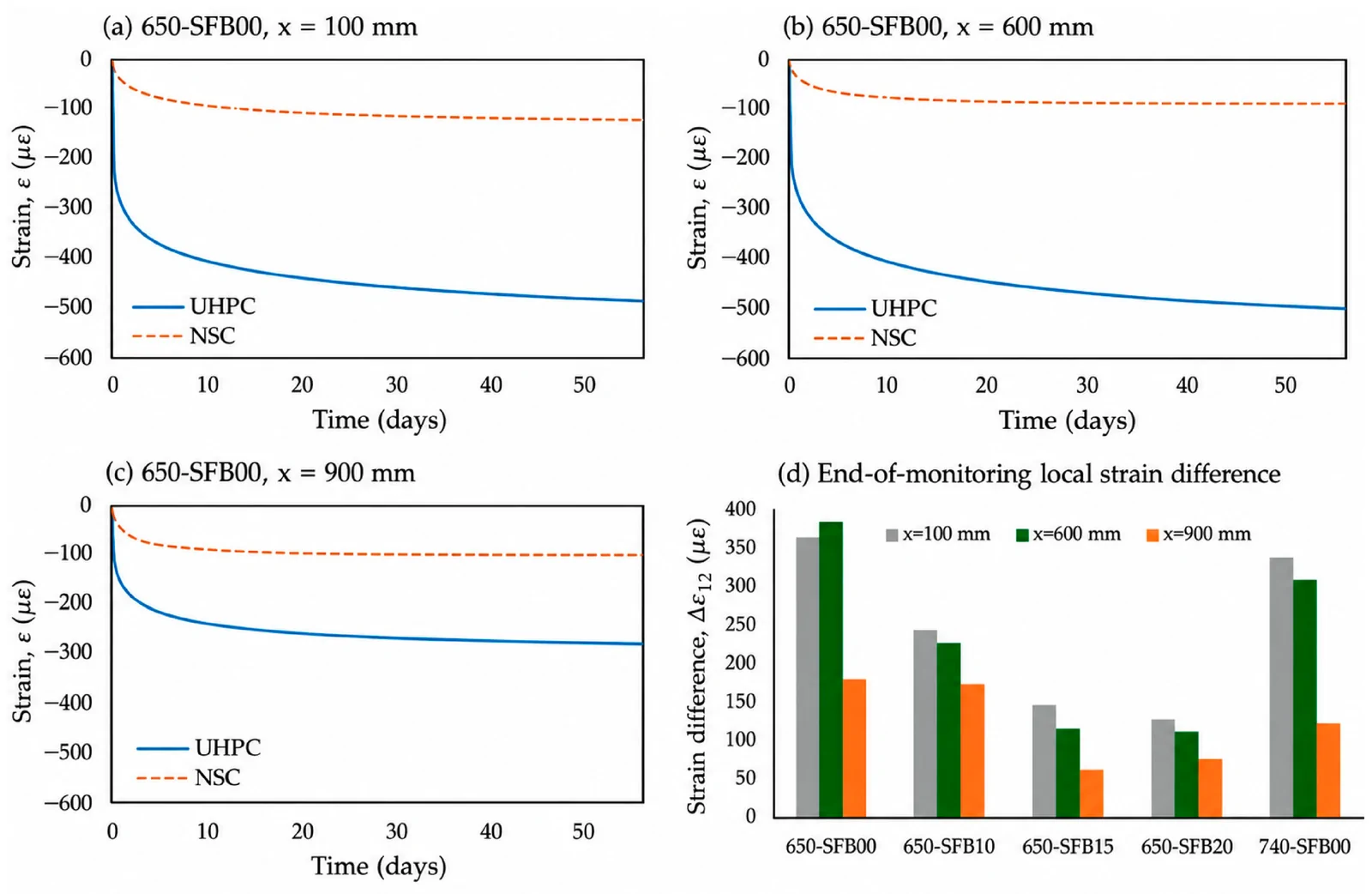
Processed strain response of the UHPC overlay and NSC substrate and the resulting local strain difference: (**a**–**c**) fitted *ε*_1_ and *ε*_2_ histories for 650-SFB00 at *x* = 100, 600, and 900 mm, respectively; (**d**) end-of-monitoring Δ*ε*_12_ at the three measurement stations for all five specimens.

Local strain difference, Δ*ε*_12_, varied with both measurement station and mixture. At *x* = 600 mm, the end-of-monitoring values were approximately 380 and 306 µε for 650-SFB00 and 740-SFB00, respectively, compared with 223, 113, and 109 µε for 650-SFB10, 650-SFB15, and 650-SFB20. Within the 650 series, Δ*ε*_12_ decreased with increasing fiber content. Processed UHPC and NSC strain histories, together with the end-of-monitoring values of Δ*ε*_12_ at all three measurement stations, are presented in Figure 7.

At *x* = 900 mm, the smallest end-of-monitoring Δ*ε*_12_ was observed in all five specimens. This station also exhibited the highest *K_r_* at the end of monitoring, as discussed in Section 4.3, indicating distinct spatial variations in the two indices. Because Δ*ε*_12_ is based on local strains measured at different depths, it is interpreted as a local strain-difference index rather than as interfacial slip.

Spatial variation in Δ*ε*_12_ also differed among the specimens. Based on the end-of-monitoring values, its range across the three measurement stations was approximately 206, 66, 81, 52, and 216 µε for 650-SFB00, 650-SFB10, 650-SFB15, 650-SFB20, and 740-SFB00, respectively. Relatively large ranges for 650-SFB00 and 740-SFB00 further illustrate that a single measurement station does not necessarily characterize the local strain difference throughout the specimen.

### 4.3. Location Dependence of the Degree of Restraint

**Figure 8:**
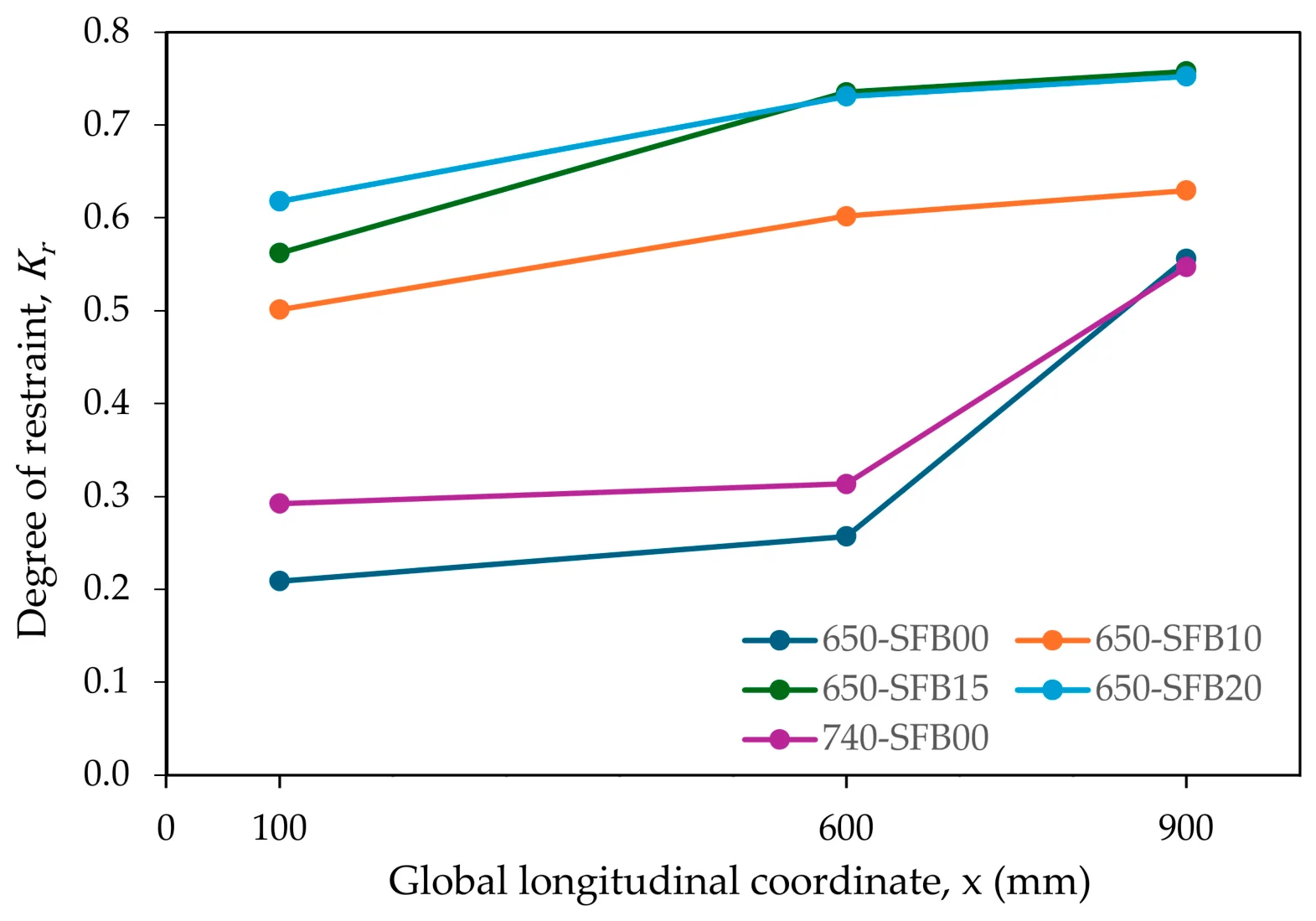
End-of-monitoring degree of restraint, *K_r_*, at *x* = 100, 600, and 900 mm.

Degree of restraint, *K_r_*, showed a clear dependence on measurement location (Figure 8). At the end of monitoring, *x* = 900 mm exhibited the highest *K_r_* in all five specimens, with values of approximately 0.556, 0.629, 0.758, 0.752, and 0.547 for 650-SFB00, 650-SFB10, 650-SFB15, 650-SFB20, and 740-SFB00. This spatial pattern was consistently observed from approximately 14 days onward.

Spatial variation in *K_r_* also differed among the specimens. Differences among the three measurement stations were more pronounced for 650-SFB00 and 740-SFB00, whereas the three fiber-reinforced specimens showed comparatively smaller spatial differences at the end of monitoring. Overall, the measured degree of restraint was not spatially uniform along the composite specimens.

### 4.4. Compatibility of Forces Inferred from Local Strains

**Figure 9:**
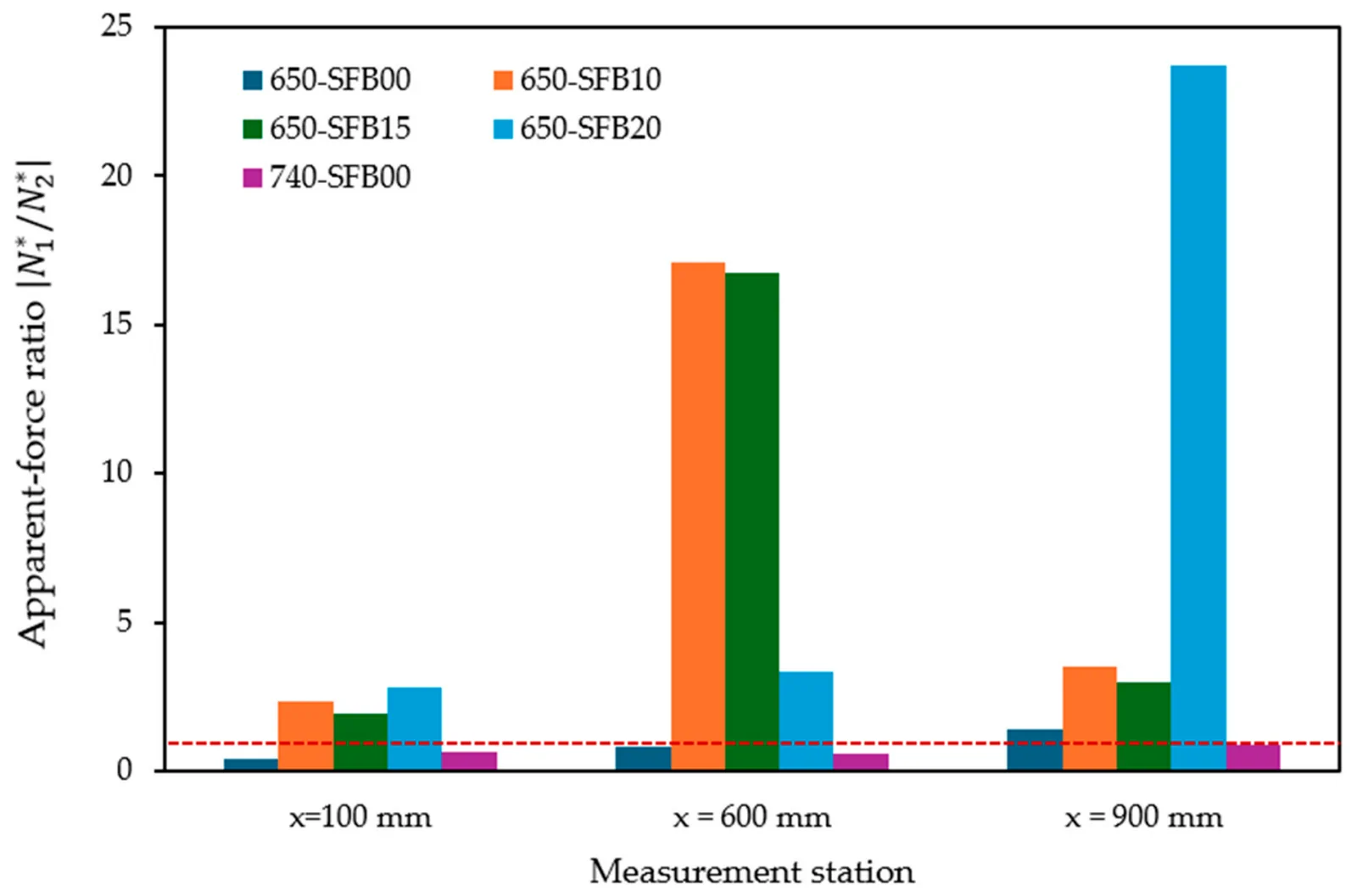
Apparent-force ratio $|N_{1}^{*}/N_{2}^{*}|$ at the end of monitoring (approximately 56 days).

The end-of-monitoring apparent-force ratios, $|N_{1}^{*}/N_{2}^{*}|$, are compared in Figure 9, with unity representing the compatibility condition of the self-equilibrated axial reference model. The ratios differed substantially from unity, indicating that the locally inferred forces did not consistently satisfy the axial reference condition. At x = 600 mm, $|N_{1}^{*}/N_{2}^{*}|$ reached approximately 17.06, 16.75, and 3.33 for 650-SFB10, 650-SFB15, and 650-SFB20, respectively; the largest ratio for 650-SFB20 instead occurred at x = 900 mm (23.70). For each of these three fiber-reinforced mixtures, the ratio between its maximum and minimum $|N_{1}^{*}/N_{2}^{*}|$ values across the three measurement stations was approximately 7.3, 8.8, and 8.4, respectively. In contrast, the two fiber-free mixtures, 650-SFB00 and 740-SFB00, remained below unity at x = 600 mm at the end of monitoring, as shown in Figure 9. The largest apparent-force imbalances were therefore observed at midspan for 650-SFB10 and 650-SFB15, but at the x = 900 mm station for 650-SFB20. Thus, the location of the largest apparent-force imbalance differed among the fiber-reinforced specimens; no single station was critical for all three. These apparent-force ratios are diagnostic measures derived from local strains; their departure from unity does not imply a violation of global force equilibrium. They are particularly sensitive to small NSC strain magnitudes in the denominator. The station-specific ratios must therefore be interpreted alongside the underlying strains and cannot identify a particular interface or support mechanism. The ratios shown in Figure 9 were calculated using the original Weibull trend fits. The dashed line at unity denotes the apparent-force compatibility condition of the self-equilibrated axial reference model; it does not indicate a failure threshold.

These results indicate that the measured local strains were not consistently compatible with their interpretation as section-representative mean axial strains within the reference model. The variation among measurement stations shows that this inconsistency depends on where the local strains are measured.

### 4.5. Comparison of Midspan Kᵣ with the Model-Based Restraint Ceiling

The model-based restraint ceiling, *K_r_*_,max_, ranged from 0.702 to 0.714 among the five mixtures. The corresponding values and measured midspan results are summarized in Table 4. Comparisons were restricted to *t* ≥ 1 day to avoid the small-denominator region associated with *ε*_free_ at very early ages.

**Figure 10:**
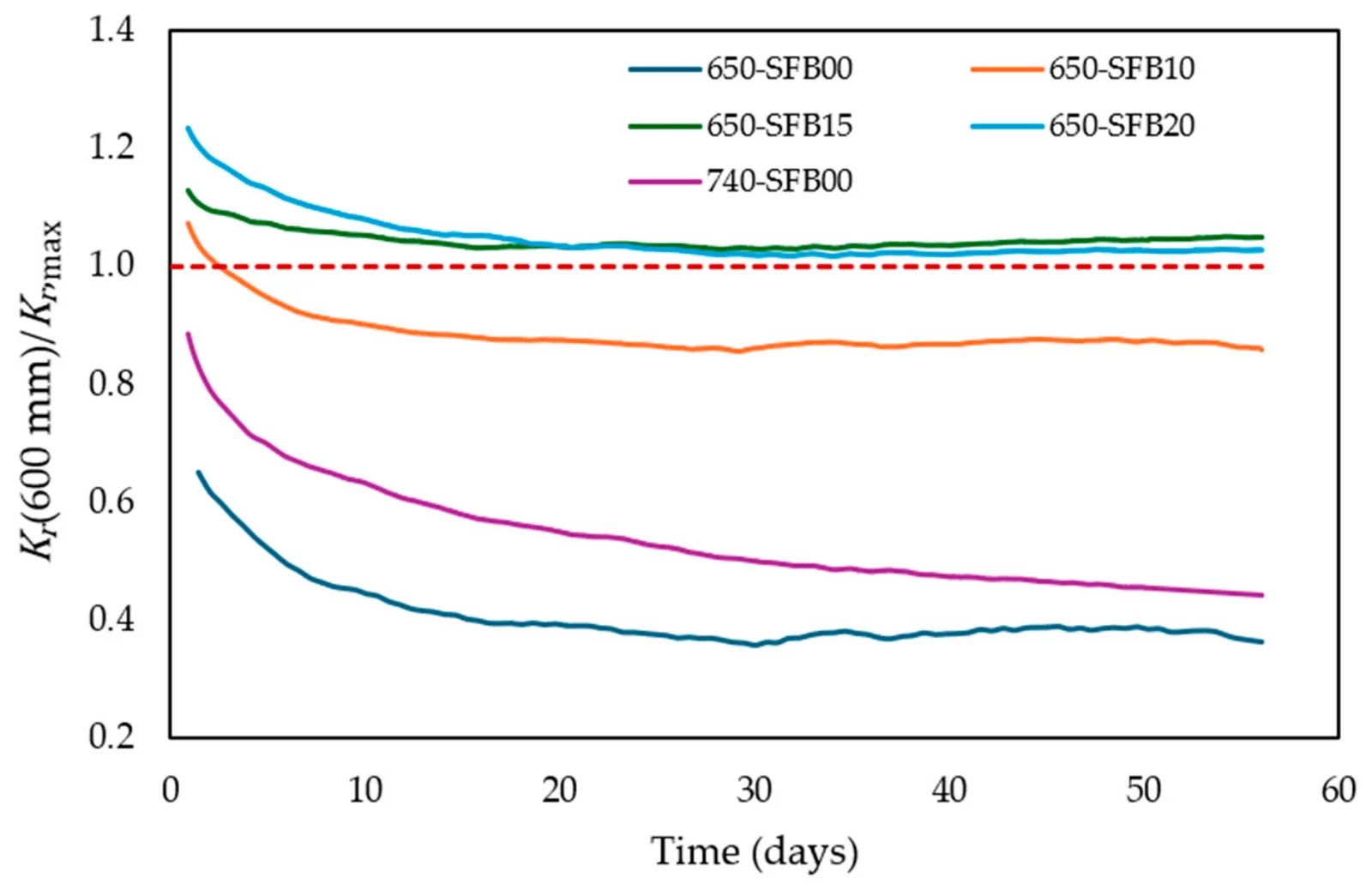
Time histories of *K_r_*(600 mm)/*K_r_*_,max_ from 1 day onward. The reference line at 1 represents the model-based restraint ceiling.

From day 1 onward, the evaluated restraint indices for 650-SFB00 and 740-SFB00 remained below *K_r_*_,max_, whereas 650-SFB10 exceeded the ceiling only briefly, until approximately 2.5 days. In contrast, the evaluated restraint indices for 650-SFB15 and 650-SFB20 remained above *K_r_*_,max_. From 28 days onward, the exceedance was approximately 2.91–4.67% for 650-SFB15 and 1.53–2.66% for 650-SFB20. The corresponding time histories are shown in Figure 10.

### 4.6. Elastic-Equivalent Tensile-Demand Ratio

**Figure 11:**
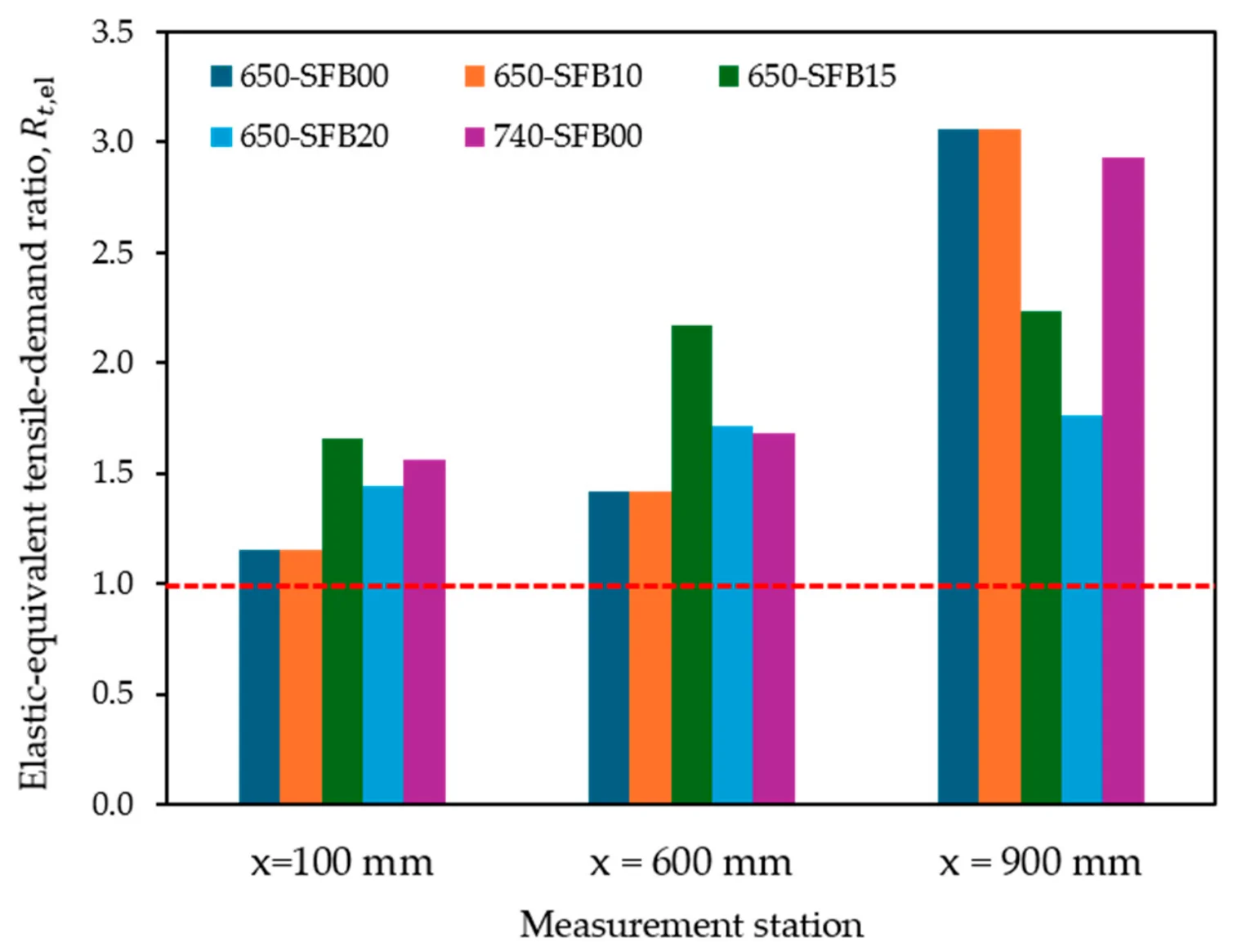
Elastic-equivalent tensile-demand ratio *R_t_*_,el_ at the end of monitoring. The reference line at *R_t_*_,el_ = 1 corresponds to the measured peak direct tensile strength, *f_t_*_,peak_.

The end-of-monitoring elastic-equivalent tensile-demand ratios, *R_t_*_,el_, are presented in Figure 11. *R_t_*_,el_ exceeded 1 at all three measurement stations for all five mixtures, with values ranging from approximately 1.15 to 3.06. The highest value occurred at *x* = 900 mm for 650-SFB00 (≈3.06), followed by 740-SFB00 at the same station (≈2.93).

No visible cracking was observed in the UHPC overlays during the 56-day monitoring period despite *R_t_*_,el_ > 1 at all evaluated mixture–location combinations. Thus, exceedance of unity in *R_t_*_,el_ did not correspond directly to visible cracking in the present specimens.

## 5. Discussion

### 5.1. Mechanical Meaning of Local Strain Measurements

The measured indices describe different aspects of the composite response. Δ*ε*_12_ represents the difference between local UHPC and NSC strains, *K_r_* quantifies the reduction in local UHPC shrinkage relative to free shrinkage, and the apparent forces $N_{1}^{*}$ and $N_{2}^{*}$ assess whether the measured local strains are consistent with their interpretation as section-representative mean axial strains.

The apparent-force analysis showed substantial departures from the self-equilibrated axial reference, particularly for the fiber-reinforced mixtures, together with pronounced variation among measurement locations. A uniform error in elastic stiffness could alter the inferred force magnitude but would not, by itself, explain this spatial variation. The measured local strains therefore cannot, in general, be treated as section-representative mean axial strains within the assumptions of the reference model.

Such behavior is mechanically plausible in a one-sided bonded overlay, where curvature, sensor eccentricity, and nonuniform interfacial load transfer can cause local strain to differ from mean axial strain [6,7,10,20,21,23]. Because only one measurement depth was available in each layer, these effects cannot be separated. Similarly, Δ*ε*_12_ should be interpreted as a local strain difference rather than as interfacial slip.

### 5.2. Model-Based Restraint Ceiling and Spatial Sensitivity

The self-equilibrated axial model provides a mechanical reference for interpreting *K_r_*, rather than a universal physical limit. The sustained exceedance of *K_r_*_,max_ for 650-SFB15 and 650-SFB20 indicates departure from the simplified axial reference, although the late-age exceedance was modest. It does not, by itself, establish an additional restraint mechanism. Because the exceedance fractions were obtained from the fitted histories, small exceedances should not be regarded as precise experimental threshold crossings. The independent comparison with the raw sensor strains at 56 days supports the interpretation of the late-age spatial pattern. However, this single-time comparison cannot establish whether the first threshold-crossing time is insensitive to fitting.

Within the 650 series, free shrinkage decreased across the tested specimens as nominal fiber content increased, a trend consistent with previous observations for steel-fiber-reinforced UHPC [30]. However, the aggregate proportions also varied within the 650 series (Table 1); the observed shrinkage trend therefore should not be attributed to fiber content alone. The degree of restraint depends on both free shrinkage and local restrained strain and therefore represents the composite response rather than a material property attributable to fiber content alone.

The spatial variation further supports this interpretation. From approximately 14 days onward, *x* = 900 mm exhibited the highest *K_r_* in all five specimens. Its proximity to the support-contact region suggests sensitivity to specimen geometry and boundary conditions, although support reactions, friction, curvature, and interface slip were not independently measured. Similar sensitivity to restraint configuration, spatial variation, and interfacial slip has been reported for UHPC–concrete systems [22,23]. Accordingly, *K_r_* is most appropriately interpreted as a local, position- and time-dependent response index rather than as an intrinsic interface property or a unique section-level restraint state.

### 5.3. Elastic-Equivalent Tensile Demand and the Absence of Visible Cracking

At the end of monitoring, *R_t_*_,el_ > 1 at all 15 mixture–location combinations, whereas no visible surface cracking was observed. The absence of visible cracking despite these elastic-equivalent ratios indicates that directly converting the local strain difference from free shrinkage into linear-elastic stress does not establish the actual tensile stress in the bonded overlay. Time-dependent UHPC behavior, including creep, further cautions against equating an elastic strain-based demand with the actual stress state [31]. The tensile response of PE-UHPFRC under imposed shrinkage has also been examined using restrained tests [32], providing a relevant comparison while involving a different fiber system from the steel-fiber UHPC investigated here.

Creep and relaxation, age-dependent material properties, curvature, and stress redistribution may contribute to this discrepancy [5,8,12,27,31]. Restrained-concrete ring testing has shown that stress relaxation affects the residual stress developed under shrinkage restraint [28]. The present measurements do not permit the separate contributions of these mechanisms to be quantified. Visual inspection also cannot exclude very fine cracks or internal damage. *R_t_*_,el_ is therefore interpreted as a normalized reference quantity rather than as a cracking or failure criterion. Together with the apparent-force analysis, this result cautions against direct conversion of local measured strain into section-level stress.

### 5.4. Implications for Shrinkage-Restraint Assessment

The results highlight the importance of reporting restraint indices together with measurement position and depth. Multi-depth strain or independent curvature measurements would allow axial and bending contributions to be separated, while quantitative stress prediction would additionally require information on time-dependent material response, interface load transfer, and boundary conditions [22,23]. For UHPC–NSC repair systems, previous studies show that substrate moisture condition, prepared-surface roughness, and bond characteristics can influence interfacial performance and composite load transfer [15,16,17,26]. These findings reinforce the need to interpret restraint indices derived from local strains within their spatial and temporal context rather than as unique specimen or interface properties.

### 5.5. Limitations and Future Work

The principal limitation is that only one strain-measurement depth was available in each layer. Curvature, interface slip, support reactions, age-dependent material properties, creep/relaxation, residual shrinkage of the mature NSC substrate, and complete environmental histories were not independently measured. In addition, the 3D surface scan documented the prepared interface geometry but was not used to calibrate a bond–slip or interface-stiffness law. Studies of UHPC repair materials cast on NSC have specifically examined interfacial bond performance and the characteristics of the interfacial transition zone [18]. The present data can therefore assess consistency with the axial reference but cannot uniquely identify the mechanisms responsible for departures from it. Additional limitations include potential in-plane fiber alignment within the 50 mm overlay, unmeasured local fiber distribution, gauge-installation eccentricity, interface-roughness variability, and the absence of reinforcing bars in the tested overlay. Fiber geometry and orientation can influence crack-bridging and tensile response [29], whereas conventional reinforcement can alter internal restraint and crack development [33]. These studies provide context but do not quantify those effects for the present specimens.

Only one composite and one free-shrinkage specimen were monitored for each mixture; trends within the 650 series should therefore be regarded as observations among the tested specimens rather than statistical estimates of mixture effects. Future work should combine multi-depth strain measurements with independent curvature and interface-slip measurements, supported by numerical sensitivity analyses to distinguish axial, bending, interface-transfer, and boundary contributions. Cohesive finite-element formulations developed for NSC–HPFRC/UHPFRC composite members provide one suitable framework for such future cracking/debonding analyses [34]. Uncertainty associated with elastic modulus, sensor eccentricity, interface geometry, and boundary restraint cannot be separated quantitatively with the present one-depth instrumentation. The observed spatial variation therefore demonstrates the limits of treating local strains as section-representative measurements, without identifying one mechanism as its cause.

## 6. Conclusions

This study examined the mechanical interpretation of shrinkage restraint in bonded UHPC–NSC overlays using simultaneous local strain measurements and a self-equilibrated axial reference model. The main conclusions are:

- Within the 650 series, free shrinkage decreased across the tested specimens as nominal fiber content increased, from approximately −616 µε for 650-SFB00 to −427 µε for 650-SFB20 at the end of monitoring; however, aggregate proportions also varied within this series.
- The measured degree of restraint, *K_r_*, showed clear spatial dependence. From approximately 14 days onward, *x* = 900 mm exhibited the highest *K_r_* in all five specimens, indicating that *K_r_* depends on measurement position and specimen configuration. The precise onset of this ordering may be affected by the trend-fitting procedure.
- The model-based ceiling *K_r_*_,max_ ranged from 0.702 to 0.714. Sustained exceedance occurred for 650-SFB15 and 650-SFB20, indicating departure from the simplified axial reference rather than evidence of an additional restraint mechanism.
- The measured local strains were not consistently compatible with their interpretation as section-representative mean axial strains. Apparent-force ratios reached approximately 17.06 and 16.75 at midspan for 650-SFB10 and 650-SFB15, respectively, whereas the largest ratio for 650-SFB20 was approximately 23.70 at x = 900 mm. The location-dependent apparent-force imbalance shows that the measured local strains cannot consistently be used as mean axial strains to infer section-level forces under the reference model.
- *R_t_*_,el_ ranged from approximately 1.15 to 3.06 and exceeded 1 at all 15 mixture–location combinations, while no visible surface cracking was observed. Direct elastic conversion of local strain should therefore not be interpreted as the actual tensile stress in the overlay.

Overall, *K_r_* is most appropriately interpreted as a local, position- and time-dependent response index. Section-level restraint and internal stress require additional information on section kinematics, time-dependent material response, interface behavior, and boundary conditions.

## Figures and Tables

**Table 1:** UHPC mixture proportions.

Component	650-SFB00	650-SFB10	650-SFB15	650-SFB20	740-SFB00
Cement (kg/m^3^)	650	650	650	650	740
Silica fume (kg/m^3^)	65	65	65	65	74
Silica powder (kg/m^3^)	130	130	130	130	148
GGBS (kg/m^3^)	130	130	130	130	148
Water (kg/m^3^)	155.9	155.9	155.9	155.9	177.5
Superplasticizer (kg/m^3^)	11.1	11.1	11.1	11.1	12.6
Steel fiber (kg/m^3^)	0	78	117	156	0
Coarse aggregate (kg/m^3^)	703	551	541	401	0
Quartz sand (kg/m^3^)	638	749	742	856	1091
Steel fiber (vol.%)	0	1.0	1.5	2.0	0

**Table 2:** Mechanical properties of the UHPC mixtures.

Mixture	Fiber (Vol.%)	f_t,peak_ (MPa)		E (GPa)	
		Mean ± SD	CoV (%)	Mean ± SD	CoV (%)
650-SFB00	0	5.49 ± 0.264	4.81	49.00 ± 0.931	1.90
650-SFB10	1.0	7.86 ± 0.528	6.72	50.90 ± 1.578	3.10
650-SFB15	1.5	7.91 ± 0.468	5.91	50.80 ± 0.814	1.60
650-SFB20	2.0	8.78 ± 0.668	7.61	48.10 ± 1.827	3.80
740-SFB00	0	6.00 ± 0.320	5.33	49.50 ± 1.188	2.40

**Table 3:** Parameters of the original Weibull-type fits for UHPC strain histories at three measurement stations. Parameters of the original Weibull-type fits for NSC strain histories at three measurement stations.

Mixture	Location	A (µε)	b	c	R^2^
650-SFB00	x = 100 mm	−612.700	−0.644	0.224	0.963
	x = 600 mm	−567.235	−0.567	0.265	0.961
	x = 900 mm	−299.819	−0.697	0.310	0.944
650-SFB10	x = 100 mm	−325.595	−0.862	0.229	0.920
	x = 600 mm	−281.227	−0.511	0.303	0.962
	x = 900 mm	−256.670	−0.473	0.335	0.969
650-SFB15	x = 100 mm	−201.710	−0.441	0.685	0.986
	x = 600 mm	−148.140	−0.520	0.306	0.958
	x = 900 mm	−134.660	−0.502	0.320	0.985
650-SFB20	x = 100 mm	−190.000	−0.411	0.399	0.987
	x = 600 mm	−127.040	−0.269	0.553	0.989
	x = 900 mm	−125.180	−0.452	0.362	0.983
740-SFB00	x = 100 mm	−512.370	−0.433	0.401	0.984
	x = 600 mm	−517.468	−0.493	0.338	0.985
	x = 900 mm	−324.885	−0.436	0.406	0.987
650-SFB00	x = 100 mm	−152.028	−0.329	0.411	0.985
	x = 600 mm	−80.000	−0.620	0.420	0.986
	x = 900 mm	−100.000	−0.580	0.520	0.989
650-SFB10	x = 100 mm	−66.620	−0.568	0.257	0.971
	x = 600 mm	−10.220	−0.407	0.387	0.902
	x = 900 mm	−52.070	−0.402	0.391	0.977
650-SFB15	x = 100 mm	−71.850	−0.530	0.298	0.986
	x = 600 mm	−10.236	−0.453	0.361	0.974
	x = 900 mm	−57.710	−0.378	0.431	0.983
650-SFB20	x = 100 mm	−49.340	−0.651	0.210	0.934
	x = 600 mm	−49.340	−0.651	0.210	0.910
	x = 900 mm	−6.414	−0.419	0.391	0.860
740-SFB00	x = 100 mm	−132.900	−0.542	0.379	0.988
	x = 600 mm	−181.578	−0.623	0.210	0.986
	x = 900 mm	−205.092	−0.553	0.299	0.985

**Table 4:** Comparison of the measured midspan degree of restraint with the model-based restraint ceiling.

Mixture	*K_r_*_,max_	End-of-Monitoring *K_r_* at x = 600 mm	Fraction of Evaluated Points Above Ceiling, t ≥ 1 d
650-SFB00	0.710	0.257	0%
650-SFB10	0.702	0.602	2.81%
650-SFB15	0.703	0.735	100%
650-SFB20	0.714	0.731	100%
740-SFB00	0.708	0.313	0%

## Data Availability

The original contributions presented in this study are included in the article. Further inquiries can be directed to the corresponding author.

## References

[1] Abellán-García J., Carvajal-Muñoz J.S., Ramírez-Munévar C. (2024). Application of ultra-high-performance concrete as bridge pavement overlays: Literature review and case studies. Constr. Build. Mater..

[2] Haber Z.B., Munoz J.F., De la Varga I., Graybeal B.A. (2018). Bond characterization of UHPC overlays for concrete bridge decks: Laboratory and field testing. Constr. Build. Mater..

[3] Xie T., Fang C., Mohamad Ali M.S., Visintin P. (2018). Characterizations of autogenous and drying shrinkage of ultra-high performance concrete (UHPC): An experimental study. Cem. Concr. Compos..

[4] Silfwerbrand J. (1997). Stresses and strains in composite concrete beams subjected to differential shrinkage. ACI Struct. J..

[5] Beushausen H., Alexander M.G. (2006). Failure mechanisms and tensile relaxation of bonded concrete overlays subjected to differential shrinkage. Cem. Concr. Res..

[6] Beushausen H., Alexander M.G. (2007). Localised strain and stress in bonded concrete overlays subjected to differential shrinkage. Mater. Struct..

[7] Zhou J., Ye G., Schlangen E., van Breugel K. (2008). Modelling of stresses and strains in bonded concrete overlays subjected to differential volume changes. Theor. Appl. Fract. Mech..

[8] Zhu Y., Zhang Y., Hussein H.H., Liu J., Chen G. (2020). Experimental study and theoretical prediction on shrinkage-induced restrained stresses in UHPC-RC composites under normal curing and steam curing. Cem. Concr. Compos..

[9] Teng L., Valipour M., Khayat K.H. (2021). Design and performance of low shrinkage UHPC for thin bonded bridge deck overlay. Cem. Concr. Compos..

[10] Guo W., Wei Y., Ma L. (2022). Shrinkage-induced warping of UHPC overlay cast on hardened NSC substrate under various conditions. Cem. Concr. Compos..

[11] Feng H., Zhang Y., Xin H., Zhao J., Shah S.Y. (2025). Restrained Shrinkage in Ultra-High-Performance Concrete (UHPC)-Normal strength concrete interface. J. Build. Eng..

[12] Liu C., Shen D., Yang X., Shao H., Tang H., Cai L. (2023). Early-age properties and shrinkage induced stress of ultra-high-performance concrete under variable temperature and uniaxial restrained condition. Constr. Build. Mater..

[13] Feng S., Xiao H., Ma M., Zhang S. (2021). Experimental study on bonding behaviour of interface between UHPC and concrete substrate. Constr. Build. Mater..

[14] Lyu J., Feng S., Zhang Q., Xiao H. (2024). Review of factors affecting bond strength between ultra-high-performance concrete and normal strength substrate. Constr. Build. Mater..

[15] Zhang Y., Zhu P., Liao Z., Wang L. (2020). Interfacial bond properties between normal strength concrete substrate and ultra-high performance concrete as a repair material. Constr. Build. Mater..

[16] Valikhani A., Jahromi A.J., Mantawy I.M., Azizinamini A. (2020). Experimental evaluation of concrete-to-UHPC bond strength with correlation to surface roughness for repair application. Constr. Build. Mater..

[17] Farzad M., Shafieifar M., Azizinamini A. (2019). Experimental and numerical study on bond strength between conventional concrete and Ultra High-Performance Concrete (UHPC). Eng. Struct..

[18] Kothari A., Rajczakowska M., Buasiri T., Habermehl-Cwirzen K., Cwirzen A. (2020). Eco-UHPC as Repair Material—Bond Strength, Interfacial Transition Zone and Effects of Formwork Type. Materials.

[19] Liu Y., Wei Y., Ma L., Wang L. (2022). Restrained shrinkage behavior of internally-cured UHPC using calcined bauxite aggregate in the ring test and UHPC-concrete composite slab. Cem. Concr. Compos..

[20] Newmark N.M., Siess C.P., Viest I.M. (1951). Tests and analysis of composite beams with incomplete interaction. Proc. Soc. Exp. Stress Anal..

[21] Girhammar U.A., Gopu V.K.A. (1993). Composite beam-columns with interlayer slip—Exact analysis. J. Struct. Eng..

[22] Hu A., Du J. (2026). Experimental study and theoretical analysis on shrinkage properties of UHPC under different constraint conditions. Int. J. Concr. Struct. Mater..

[23] Lyu J., Feng S., Qiu H., Shao C., Duan S., Li R., Guo S., Zhang Q. (2026). Influence of shrinkage of UHPC overlays on bond restrained stress and bond strength of UHPC-NSC. Structures.

[24] Guo W., Wei Y., Ma L., Liu Y., Guo R. (2023). Effect of grouped L-shape rebar connectors on the shrinkage behavior of UHPC overlay cast on hardened NSC substrate. Constr. Build. Mater..

[25] Wei Y., Guo W., Ma L., Liu Y., Yang B. (2023). Materials, structure, and construction of a low-shrinkage UHPC overlay on concrete bridge deck. Constr. Build. Mater..

[26] Zhang B., Yu J., Chen W., Chen J., Li H., Niu J. (2024). Interface Shear Failure Behavior Between Normal Concrete (NC) and Ultra-High Performance Concrete (UHPC). Int. J. Concr. Struct. Mater..

[27] Yang G., Zhao H., Hu Z., Zhang W., Xiang Y., Jin M., Wan-Wendner R., Liu J. (2025). Prediction of restrained stress for UHPC: Considering relationship between long-term and in-situ creep. Constr. Build. Mater..

[28] Hossain A.B., Weiss J. (2004). Assessing residual stress development and stress relaxation in restrained concrete ring specimens. Cem. Concr. Compos..

[29] Yoo D.-Y., Kim S., Park G.-J., Park J.-J., Kim S.-W. (2017). Effects of fiber shape, aspect ratio, and volume fraction on flexural behavior of ultra-high-performance fiber-reinforced cement composites. Compos. Struct..

[30] Wu Z., Shi C., Khayat K.H. (2019). Investigation of mechanical properties and shrinkage of ultra-high performance concrete: Influence of steel fiber content and shape. Compos. Part B Eng..

[31] Huang Y., Wang J., Wei Q., Shang H., Liu X. (2023). Creep behaviour of ultra-high-performance concrete (UHPC): A review. J. Build. Eng..

[32] Hajiesmaeili A., Hafiz M.A., Denarié E. (2021). Tensile response of Ultra High Performance PE Fiber Reinforced Concretes (PE-UHPFRC) under imposed shrinkage deformations. Mater. Struct..

[33] Feng Z., Li C., Yoo D.-Y., Pan R., He J., Ke L. (2021). Flexural and cracking behaviors of reinforced UHPC beams with various reinforcement ratios and fiber contents. Eng. Struct..

[34] Savino V., Lanzoni L., Tarantino A.M., Viviani M. (2020). A cohesive FE model for simulating the cracking/debonding pattern of composite NSC-HPFRC/UHPFRC members. Constr. Build. Mater..

